# Nora Virus VP4b and *ORF1* Circulate in Hemolymph of Infected *D. melanogaster* with Coordinate Expression of Vago and Vir-1

**DOI:** 10.3390/vaccines8030491

**Published:** 2020-08-31

**Authors:** Amanda Macke, Wilfredo Lopez, Darby J. Carlson, Kimberly A. Carlson

**Affiliations:** Department of Biology, University of Nebraska at Kearney, Kearney, NE 68849, USA; a26macke@gmail.com (A.M.); lopez.w.alex@gmail.com (W.L.); carlsondj@unk.edu (D.J.C.)

**Keywords:** Nora virus, Vago, Vir-1, hemolymph, *Drosophila melanogaster*, picorna-like virus

## Abstract

Study of the novel RNA virus, Nora virus, which is a persistent, picorna-like virus that replicates in the gut of *Drosophila melanogaster* offers insight into human innate immunity and other picorna-like viruses. Nora virus infection leads to a locomotor abnormality and upregulation of two candidate target proteins, Vago and Virus-induced RNA 1 (Vir-1). These proteins are uncharacterized in response to Nora virus. We hypothesize that Nora virus is circulating in the hemolymph of Nora virus-infected *D. melanogaster*, allowing for migration beyond the primary site of replication in the gut. Analysis by qRT-PCR demonstrated biphasic viral load and corresponding *vago* and *vir-1* transcription levels, suggesting transcription of *vago* and *vir-1* occurs in response to viral infection. However, Vir-1 is also present in virus-free *D. melanogaster* suggesting basal expression or alternative functions. Presence of Nora virus RNA and the Viral Protein 4b (VP4b), in hemolymph of infected *D. melanogaster* supports the hypothesized circulation of Nora virus in the hemolymph. The study suggests that impaired locomotor function may be due to transport of Nora virus from the gut to the brain via the hemolymph.

## 1. Introduction

The *Drosophila melanogaster* innate immune system shares several characteristics with its mammalian counterparts, including origin, type, and timeframe of the immune response [1]. Of the genes associated with the innate immune response in *D. melanogaster,* 77% are shared with humans [2]. For this reason, *D. melanogaster* provide an easily manipulated model for many immune pathways and offer novel target proteins for study in disease models. One group of mammalian proteins, the Toll-like receptor family, was first identified in response to a *Drosophila* protein, Toll, involved in activation of the Nuclear factor-Kappa B (NF-κB) pathway [3]. Further, the NF-κB pathway in *Drosophila* continues to be studied to better understand this pathway in mammalian models. In *D. melanogaster,* innate immune responses include autophagy, RNA interference (RNAi), and several pathways including the Toll, immune deficiency (IMD), and Janus kinase/signal transducers and activators of transcription (Jak-STAT) pathways [4]. Autophagy is a cytoplasmic degradation process, involved in starvation and stress responses, development, cell death, and immune responses. *Drosophila* autophagy relies upon the stimulation of pattern-recognition receptors inducing antimicrobial peptide production and activation of the Toll and IMD pathways [5]. RNAi inhibits viral replication by fragmenting the viral genome and using these fragments, small interfering RNAs (siRNAs) to target the viral genome [6]. The siRNAs are loaded into the RNA-induced silencing complex (RISC) and serve as a guide indicating which sequences to cleave. The RNAi response leads to production of tumor necrosis factor receptor-associated factor (TRAF) [7]. The IMD pathway is implicated in the recognition of viral particles by peptidoglycan recognition proteins (PGRP-LC and PGRP-LE) and leads to the cleavage of the NF-κB transcription factor, Relish (Rel) by TRAF. The cleaved Relish promotes transcription of IMD-regulated genes, including *vago* [8]. Vago, a secretory, cytokine-like molecule serves to activate the Jak-STAT pathway in conjunction with Unpaired (Upd), and the Domeless (Dome) receptor [8,9]. Activation of the Jak-STAT pathway regulates the expression of several genes involved in modulating viral load, including *vir-1* that encodes virus-induced RNA 1 (Vir-1). Vir-1 has been implicated in response to *Drosophila* C virus (DCV) and Flock House virus (FHV), and in a potential cold tolerance role [4,10,11]. Upregulated genes in DCV-infected *D. melanogaster* included *vago* and *vir-1* with 45-fold induction of *vago* and 46-fold induction of *vir-1* [4].

These immune pathways are well understood in response to DCV, but not in response to Nora virus infection. In addition, Vir-1 and Vago response to Nora virus has not been characterized. Nora virus is a small, picorna-like virus that establishes a persistent infection in *D. melanogaster*. *D. melanogaster* Nora virus consists of four open reading frames (ORFs) [12]. *ORF1* encodes an RNA interference (RNAi) suppressor that possibly contributes to the establishment of the aforementioned persistent infection seen in *D. melanogaster* [13]. *ORF2* encodes a picorna-like replicative cassette, which consists of a helicase, protease, and RNA-dependent RNA polymerase. The capsid proteins are encoded in *ORF3* (viral protein 3; VP3) and *ORF4* (VP4 that is processed into VP4A, B, and C) [14]. As an enteric virus, it replicates primarily in the gut tissue, is localized in the intestines, and is transmitted horizontally [13]. Our lab has documented a locomotor defect with persistent Nora virus infection. Infected *D. melanogaster* are significantly hindered in terms of geotaxis, when compared to their uninfected counterparts [15]. To explain this phenotype, it is proposed that Nora virus-specific molecules, *ORF1* and VP4B, are circulating in the hemolymph to the brain. Hemocytes can process viral genetic material to produce virus-derived small interfering RNAs (vsRNAs) [16]. These vsRNAs are packaged into exosomes and taken up by surrounding cells, conferring RNAi immunity. In addition, we hypothesize that both Vago and Vir-1 are being produced in response to Nora virus infection and circulating in the hemolymph. To address the question of whether Vago and Vir-1 are produced in response to Nora virus infection, we analyzed whole fly lysates. In addition, to determine if these molecules are circulating, the hemolymph of Nora virus-infected and uninfected *D. melanogaster* was analyzed for presence of Vago, Vir-1, and Nora virus capsid protein (VP4B), as well as Nora virus transcript levels (*ORF1*). Lastly, to determine if these levels change as the fly ages, a time course analysis for these molecules was performed, using both hemolymph and whole flies. We predict that if Nora virus is circulating in the hemolymph, Vago and Vir-1 will be present to regulate viral load in Nora virus-infected *D. melanogaster*.

## 2. Materials and Methods

### 2.1. Fly Husbandry and Time Course

Witi *Rel*^E23^ (a kind gift from Dan Hultmark from Umeå, Sweden) were maintained at 25 °C on standard cornmeal, molasses, and torula yeast medium with diurnal light. Flies were either infected fecal-orally to establish Nora virus-infected stocks or kept Nora virus-free to maintain uninfected stocks for further analysis [17]. Once adequately established, stocks were expanded for fly collection by transferring flies into new bottles. The next generation of flies was allowed to eclose and virgin females were collected for further analysis. The virgin females were placed in pint cages with air ventilation, a food vial, and an access point for a mouth aspirator to add and remove flies. The following two conditions were established: Nora virus-infected flies (NV+) and Nora virus uninfected (NV−) flies [18]. Two hundred virgin female flies were collected for each condition and aged (for time point analysis). All living flies were collected from the cages at 3, 10, 17, and 20 days post-eclosion, hemolymph was extracted, the flies were placed into a microcentrifuge tube and frozen at −80 °C for future analysis. Whole fly protein and RNA were isolated from the collected flies at a later time.

### 2.2. Hemolymph Sampling

After being anesthetized, flies were punctured in the thorax using a fine tungsten needle and placed in a 0.5 mL microcentrifuge tube. This tube was punctured through the tip using a 16-gauge needle. The punctured 0.5 mL tube was placed inside a 1.5 mL microcentrifuge tube and centrifuged at 5000 rcf for 5 min at 4 °C. For each collection of hemolymph, 200 to 600 flies were punctured. For total RNA concentration, each hemolymph sample was quantitated using a NanoDrop ^TM^ 2000c spectrophotometer (ThermoFisher Scientific, Waltham, MA, USA) per manufacturer’s instructions to assess RNA purity. For protein concentration, the total protein in the hemolymph was quantitated by BCA Protein Assay (ThermoFisher Scientific) per manufacturer’s instructions. Flies were frozen at −80 °C and used for whole fly protein and RNA extractions at a later time.

### 2.3. Preparation of Mouse Antisera against Recombinant VP4b, Vago, and Vir-1 Proteins

All experimental procedures were reviewed and approved by the UNK Institutional Animal Care and Use Committee (IACUC # 091311a). Mouse antisera against the individual recombinant proteins were prepared as follows. CD-1 outbred retired breeder female mice (Charles River Laboratories, Wilmington, MA, USA) were used for the production of antisera. Mice were housed, 5 per cage, in a holding facility maintained at constant temperature (25 °C) under a 12:12 light/dark cycle. Food and water were provided ad libitum. All mice were pre-bled via the retro-orbital route. Twenty micrograms of recombinant protein were mixed with 10 mM Tris, pH 8.0, and 250 mM NaCl buffer to give a volume of 100 μL. An equal volume of Freund’s complete adjuvant (Pierce Chemical Co., Dallas, TX, USA) was added and an emulsion was formed by passing the mixture back and forth through a double-hub needle. Two hundred microliters of the emulsion containing the protein was administered to each mouse via a subcutaneous route on day one. Four weeks later, a test bleed from the mice was taken and a booster injection identical to the primary injection was given, with the exception that the inoculum was prepared using Freund’s incomplete adjuvant (Pierce Chemical Co.). Each two weeks following the booster injection, additional test bleeds were taken until the titer waned.

### 2.4. Protein Extraction and Western Blot Analysis

Total protein was extracted from 8 to 10 flies by homogenizing in RIPA lysis buffer (1% NP40; 0.1% SDS; 0.15 M NaCl; 0.01 M NaPO_4_, pH 7.2; 2 mM EDTA) with 1X Sigma protease inhibitor (Sigma-Aldrich, St. Louis, MO, USA) and centrifuged at 16,000× *g* for 10 min at 4 °C yielding a whole fly protein lysate to be further analyzed. Whole fly protein lysates were quantitated using the BCA Protein Assay (Thermofisher Scientific) per manufacturer’s instructions. Recombinant VP4b (prepared as described in [19]) and recombinant Vago and Vir-1 proteins (Genscript, Piscataway, NJ, USA) were used as positive controls during analysis. Whole fly protein lysates, hemolymph samples, and recombinant proteins were combined with Laemmli buffer with 5% 2-mercaptoethanol (Bio-Rad, Hercules, CA, USA) and boiled for 25 min. For analysis of the preliminary time course, approximately 13–49 μg of the whole fly protein lysate for days 3, 10, 17, and 24 were analyzed. A previously assessed protein lysate was used as an additional positive control for comparison. After optimization of the preliminary time course, 50–200 μg of protein sample was loaded into each lane (50 μg for VP4b, 100 μg for Vir-1, Vago and GAPDH analysis in total whole fly protein lysates; 60 μg for VP4b, 120 μg for Vir-1 and GAPDH, and 200 μg for Vago analysis in hemolymph samples) of Mini-PROTEAN TGX gels (Bio-Rad) and transferred to nitrocellulose membranes (Bio-Rad) using the Trans-Blot Turbo Transfer System (Bio-Rad) for 10 min at 25 V. Membranes were blocked using 5% nonfat milk (Nestle, Glendale, CA, USA) in 1X TBST buffer (5 M NaCl, 1 M Tris-HCl, pH 7.5, Tween-20) for thirty minutes. After blocking, the membranes were incubated with a 1:2000 dilution of VP4b antisera, 1:5000 dilution of GAPDH antibody (IMGENEX, San Diego, CA), 1:500 dilution of Vago antisera, or 1:500 dilution of Vir-1 antisera in 5% nonfat milk and TBST buffer 2 to 3 h at room temperature or overnight at 4 °C. The Vago antisera dilution was subject to change to a 1:250 dilution to increase sensitivity of antisera and later blots used the Western blot Immuno Booster reagent system (Takara Bio, Kusatsu, Shiga, Japan) to increase Vago detection. After incubation, the membranes were washed three times with 1X TBST buffer for 10 min per wash. For incubation involving the preimmune antisera, the washes were done for 30 min per wash to reduce background and cross reacting product of the antisera. Membranes were incubated with a 1:10,000 dilution of alkaline phosphatase-conjugated anti-mouse IgG serum (ThermoFisher Scientific) or a 1:10,000 dilution of alkaline phosphatase-conjugated anti-rabbit IgG serum (ThermoFisher Scientific) for 30 min to 2 h at room temperature or overnight 4 °C. After incubation, membranes were washed three times with 1X TBST buffer and once with 1X TBS (5 M NaCl and 1 M Tris-HCl, pH 7.5) buffer for 10 min per wash. The color reaction was carried out using 1-Step NBT/BCIP (ThermoFisher Scientific) for 5–20 min and stopped using distilled water.

### 2.5. RNA Extraction, RT-PCR, and qRT-PCR Analyses of Nora Virus, Vago, and Vir-1

Total RNA was extracted from 10 flies per sample using TRIzol^®^(ThermoFisher Scientific) per manufacturer’s instructions (ThermoFisher Scientific). Each sample was quantitated using a NanoDrop ^TM^ 2000c spectrophotometer (ThermoFisher Scientific) to assess RNA purity and concentration. Samples were analyzed for the presence of Nora virus using Nora *ORF1* 55–844 (Forward 5′-TGGTAGTACGCAGGTTGTGGGAAA-3′; Reverse 5′-AAGTGGCATGCTTGGCTTCTCAAC-3′) primers or presence of DCV using *DCV7/8* (Forward 5′-AGTATGATTTTGATGCAGTTGAATCTC-3′; Reverse 5′-GAAGCACGATACTTCTTCCAAACC-3′) primers and Promega Access Quick RT-PCR master mix and reverse transcriptase (Promega, Madison, WI, USA), SuperScript III One-Step RT-PCR Platinum (Invitrogen, Carlsbad, CA) or qScript XLT 1-Step RT-PCR (Quantabio, Beverly, MA, USA) according to the manufacturer’s instructions. The positive controls were an RNA extraction that previously tested positive for Nora virus or DCV. Reactions were set-up under the following conditions for Nora virus: 50 °C for 30 min, 94 °C for 2 min, (94 °C for 30 s, 55 °C for 30 s, and 68 °C for 1 min) for 30 cycles, 68 °C for 5 min, and hold at 4 °C; and DCV: 50 °C for 30 min, 94 °C for 4 min, (94 °C for 40 s, 52 °C for 40 s, and 72 °C for 1 min) for 35 cycles, 72 °C for 7 min, and hold at 4 °C. Samples were analyzed on a 0.8% agarose gel in a TAE buffer solution at 80 V for 1 h. A positive reaction yielded a product at approximately 790 bp for Nora virus and 524 bp for DCV.

TaqMan Gene Expression Assay kits (Applied Biosystems, Foster City, CA, USA) and the 7500 Real Time PCR System (Applied Biosystems) were used to perform reverse transcription quantitative PCR (qRT-PCR) according to the manufacturer’s instructions. Each reaction was set up with 200 ng of RNA. The TaqMan probe sets were *Ribosomal protein L32* (*RpL32*; endogenous control; assay #Dm02151827_g1), Nora virus *ORF1* (AIRSA9W), *vago* (assay #Dm01837827_g1), and *vir-1* (assay #Dm01807216_m1). Replicate 25 μL reactions for each of the 3 experiments were carried out under the following conditions: 48 °C for 30 min and 95 °C for 10 min (95 °C for 15 s and 60 °C for 1 min) repeated for 40 cycles. The PCR products were analyzed in the linear range for amplification with *RpL32* using the 7500 Real Time PCR System Sequence Detection Software^®^ (Applied Biosystems). The relative quantitative results in viral load using *ORF1* as the target gene were used to determine changes in gene expression on a Log_2_ scale via the ΔΔCT method [20]. For all ΔΔCT analyses, virus-free samples were used as the reference for the ddCt value. Statistical analysis was conducted using an unequal variance, two-tail Student’s *t*-test.

## 3. Results

### 3.1. Validation of Nora Virus and DCV Infection Using RT-PCR

The presence of Nora virus *ORF1* and a DCV specific gene product were analyzed by RT-PCR using gene specific primers. A 790 bp product for *ORF1* verified Nora virus infection and a 524 bp product for the DCV gene verified DCV infection. Stocks that were created to be infected with Nora virus demonstrated a 790 bp product confirming infection, but did not have a positive product for DCV, showing that these stocks were only infected with Nora virus. Virus-free (uninfected) stocks were also tested for the presence of Nora virus and DCV, and were found to be negative for both the Nora virus specific *ORF1* RT-PCR product and the DCV specific RT-PCR product.

### 3.2. Nora Virus is Present in the Hemolymph of Infected D. melanogaster

**Figure 1:**
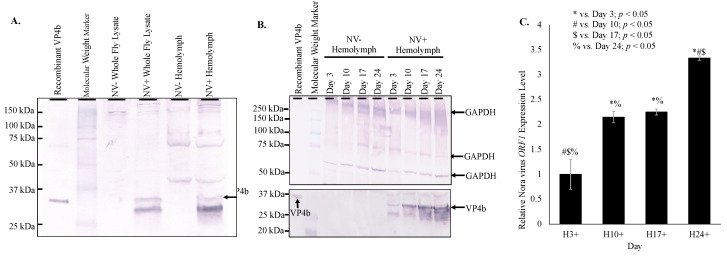
Nora virus presence in the hemolymph of infected *D. melanogaster.* (**A**). Western blot analysis of NV+ and NV− whole fly lysates and hemolymph using anti-VP4b serum as the primary antibody for detection. Arrow indicates the expected product at approximately 32 kDa. (**B**). Western blot analysis time study of NV+ and NV− hemolymph samples from time study trials. The top panel is the control that used anti-GAPDH primary antibody for detection. Arrows indicate expected GAPDH products at approximately 300, 250, and 50 kDa. The bottom panel used anti-VP4b serum as the primary antibody for detection. Arrows indicate the expected VP4b product at approximately 32 kDa. (**C**). qRT-PCR analysis of expression of Nora virus *ORF1* in hemolymph of Nora virus-infected *D. melanogaster* on days 3, 10, 17, and 24 relative to age-matched virus-free controls. The results are from 3 experiments done in triplicate. The error bars are standard error of the mean, and ^*,#,$,%^ represent statistical significance (*p* < 0.05) as denoted in the figure legend.

Nora virus components, VP4b and ORF1, were found in the hemolymph of infected *D. melanogaster* by Western blot and qRT-PCR, respectively. Western blot analysis of hemolymph from infected *D. melanogaster* demonstrated a 32 kDa product as was expected for VP4b (Figure 1A) [18]. Increasing VP4b was detected by Western blot analysis of hemolymph from days 3, 10, 17, and 24 (Figure 1B). Protein products were quantified using ImageJ (https://imagej.nih.gov/.html), demonstrating an overall increase of VP4b in hemolymph from day 3 (5.2 net increase) through day 24 (12.4 net increase; Figure 1B). Doublet products were observed in all VP4b blots in whole fly or hemolymph samples, which represented immature and mature forms of VP4b [18]. By qRT-PCR, Nora virus *ORF1* was detected at a 3.1 × 10^5^ fold increase in hemolymph of infected, day 3 *D. melanogaster* relative to Nora virus *ORF1* present in hemolymph of virus-free, day 3 *D. melanogaster* (Figure 1C).

### 3.3. Viral Load Follows a Biphasic Pattern

**Figure 2:**
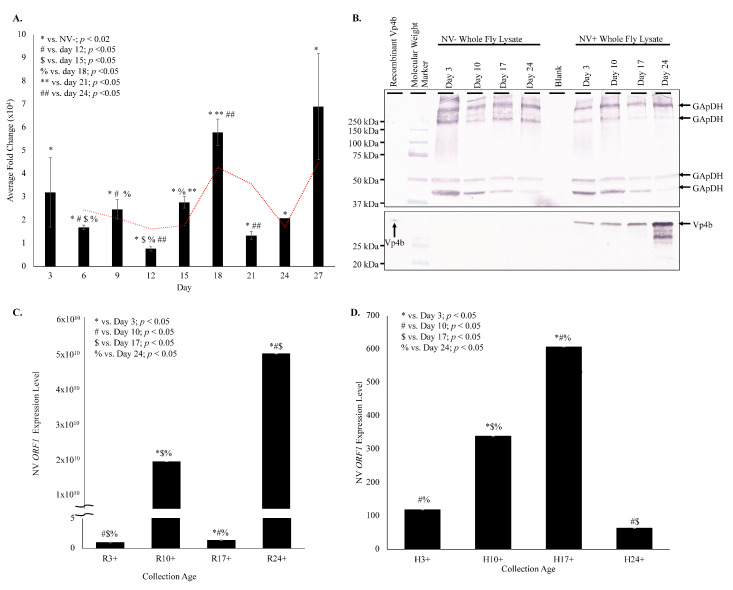
Nora virus viral load follows a biphasic pattern. (**A**). Nora virus load in whole fly samples of *D. melanogaster* infected with Nora virus (NV+; *n* = 78 flies per time point) over time (days 3, 6, 9, 12, 15, 18, 21, 24, and 27). ^*^ Viral load was significantly increased at all NV+ time points (*p* < 0.002). The ^#,$,%,^^**,##^ represent statistical significance (*p* < 0.05) for timewise comparisons as denoted in the figure legend. The red dotted line indicates the moving average over time beginning at day 6. The error bars are the standard error of the mean. (**B**). Western blot analysis of NV+ and NV− whole fly lysates over time (days 3, 10, 17, and 24). The top panel is the control that used anti-GAPDH primary antibody for detection. Arrows indicate expected GAPDH products at approximately 300, 250, 50, and 25 kDa. The bottom panel used anti-VP4b serum as the primary antibody for detection. Arrow indicates the expected VP4b product at approximately 32 kDa, which is only present in the NV + lysates. (**C**). qRT-PCR analysis of expression of Nora virus *ORF1* in whole fly lysate from Nora virus-infected *D. melanogaster* at days 3, 10, 17, and 24. The results are from 3 experiments done in triplicate. The error bars are standard error of the mean, and ^*,#,$,%^ represent statistical significance (*p* < 0.05) as denoted in the figure legend. (**D**). qRT-PCR analysis of expression of Nora virus *ORF1* in hemolymph as compared to whole fly RNA lysates from Nora virus-infected *D. melanogaster* at days 3, 10, 17, and 24 relative to Nora virus *ORF1* present in whole fly lysate of age-matched virus-free *D. melanogaster*. The results are from 3 experiments done in triplicate. The error bars are the standard error of the mean, and ^*,#,$,%^ represent statistical significance (*p* < 0.05) as denoted in the figure legend.

Nora virus load in whole fly RNA was determined by qRT-PCR at days 3, 6, 9, 12, 15, 18, 21, 24, and 27. The biphasic pattern is illustrated by the viral load fluctuating from 32,091 fold change at day 3 to 7960 fold change at day 12, and climbing to 68,952 fold change at day 27 (Figure 2A).

Products were quantified using ImageJ (https://imagej.nih.gov/.html), demonstrating an overall increase of VP4b in whole fly lysates from day 3 (1.8 net increase) through day 24 (3.5 net increase; Figure 2B). This increasing VP4b corroborates the biphasic viral load. The whole fly RNA viral load is mirrored in the hemolymph, with viral load plateauing in the hemolymph around day 17 (Figure 1C), when the whole fly viral load was low and was rising again (Figure 2C). In the hemolymph, viral load was 2.2-fold increased on day 10 relative to day 3, 2.3-fold increased on day 17 relative to day 3, and 3.3-fold increased on day 24 relative to day 3 (Figure 1C). Viral load was significantly different (*p* < 0.05) between all time points, except between days 10 and 17. Nora virus load in hemolymph relative to whole fly RNA was analyzed by qRT-PCR of Nora virus ORF1 (Figure 2D). Whole fly RNA at day 3 had a 100-fold increase in Nora virus *ORF1* relative to day 3 hemolymph, a 300-fold increase in day 10 samples, and a 60-fold increase in day 24 samples. The difference between day 3 and 10 and between day 10 and 24 were significant (*p* < 0.05).

### 3.4. Vago Expression Is Upregulated in Nora Virus-Infected D. melanogaster

**Figure 3:**
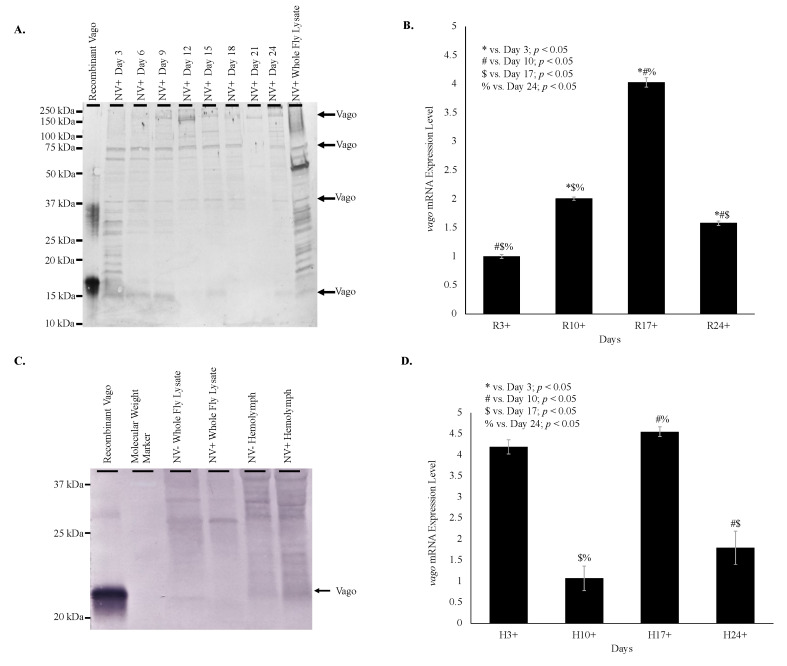
Vago expression is upregulated in Nora virus-infected *D. melanogaster*. (**A**). Western blot analysis of Nora virus-infected *D. melanogaster* over a 24-day time course in whole fly lysates using anti-Vago serum as the primary antibody for detection. Vago monomers were faintly detected at approximately 16.5 kDa for days 3, 6, 9, 15, and 24, and in the whole fly lysate samples. In addition, multiple Vago products were detected at a range of 16.5 kDa to 20 kDa for day 3 and the whole fly protein lysate for comparison. Potential Vago proteins were detected at 37 and 75 kDa for days 3, 6, 9, 12, 15, and 24. No proteins were detected at these molecular weights for day 21. In addition, potential Vago proteins were detected at 150 kDa for day 9, 12, 15, 18, 21, and 24, and in the whole fly lysate samples. (**B**). qRT-PCR analysis of expression of *vago* in whole Nora virus-infected *D. melanogaster* on days 3, 10, 17, and 24 relative to age-matched virus-free controls. The results are from 3 experiments done in triplicate. The error bars are standard error of the mean, and ^*,#,$,%^ represent statistical significance (*p* < 0.05) for timewise comparisons as denoted in the figure legend. (**C**). Western blot analysis of NV+ and NV− whole fly lysates and hemolymph using anti-Vago serum as the primary antibody for detection. Arrow indicates the expected product from recombinant Vago at approximately 22 kDa. (**D**). qRT-PCR analysis of expression of *vago* in hemolymph of Nora virus-infected *D. melanogaster* on days 3, 10, 17, and 24 relative to age-matched virus-free controls. The results are from 3 experiments done in triplicate. The error bars are standard error of the mean, and ^*,#,$,%^ represent statistical significance (*p* < 0.05) for timewise comparisons as denoted in the figure legend.

Vago was detected at 16.5 kDa in the time study in whole fly lysates from infected *D. melanogaster* on day 3, 6, 9, 15, and 24 (Figure 3A). Multiple product sizes were observed due to multimer formation. Analysis of *vago* transcription by qRT-PCR indicated increasing *vago* transcription with increasing viral load through day 17 in whole fly lysate (Figure 3B). Western blot analysis of hemolymph from infected *D. melanogaster* demonstrated a 22 kDa product for Vago (Figure 3C). Transcription of *vago* increased 2-fold in day 10, 4-fold in day 17, and 1.5-fold in day 24 relative to day 3 whole fly lysate relative to age-matched virus-free controls. The difference between all ages was significant (*p* < 0.05). Transcription of *vago* in hemolymph of infected *D. melanogaster* did not increase in day 10 but increased 4-fold on day 3 and 17 and 2-fold on day 24 relative to age-matched virus-free *D. melanogaster* (Figure 3C). The difference between days 10 and 17, 10 and 24, and 17 and 24 were significant (*p* < 0.05).

### 3.5. Vir-1 Presence Does Not Require Viral Infection

**Figure 4:**
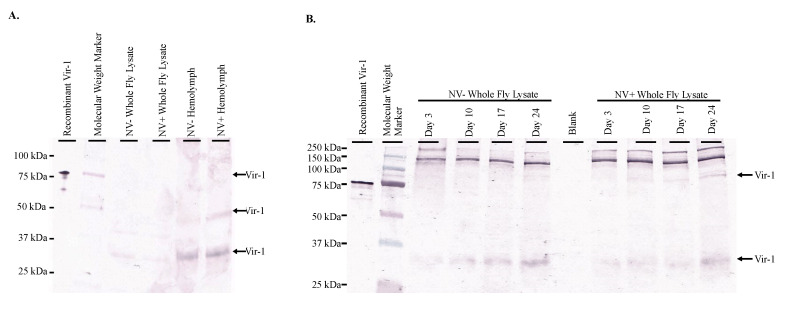
Vir-1 presence does not require viral infection. (**A**). Western blot analysis of Nora virus-infected (NV+) and virus-free (NV−) whole fly lysates and hemolymph using anti-Vir-1 serum as the primary antibody for detection. Arrows indicate products at approximately 75 kDa in the recombinant Vir-1 sample; at 50 kDa in the NV+ hemolymph sample; and at 30 kDa in NV− and NV+ whole fly lysates and NV− and NV+ hemolymph samples. (**B**). Western blot analysis of NV+ and NV− whole fly lysates over time using anti-Vir-1 serum for detection. Arrows indicate products at approximately 75 kDa in the Recombinant Vir-1 sample and NV+ whole fly lysates from days 10, 17, and 24. Arrow indicates product at 30 kDa in all sample lanes. In both panels, the products for Vir-1 expression can be seen in both infected and virus-free samples.

Western blot analysis of both whole fly lysates and hemolymph of Nora virus-infected and virus-free *D. melanogaster* yielded Vir-1 products at 30 kDa for whole fly lsyates and hemolymph samples, 50 kDa in the NV+ Hemolymph sample, and 75 kDa in the Recombinant Vir-1 lane (Figure 4A). Multiple product sizes were observed likely due to multimer formation. Vir-1 was present at all ages in both Nora virus-infected and virus-free *D. melanogaster* (Figure 4B).

### 3.6. Vir-1 Expression Is Increased in Nora Virus-Infected D. melanogaster

**Figure 5:**
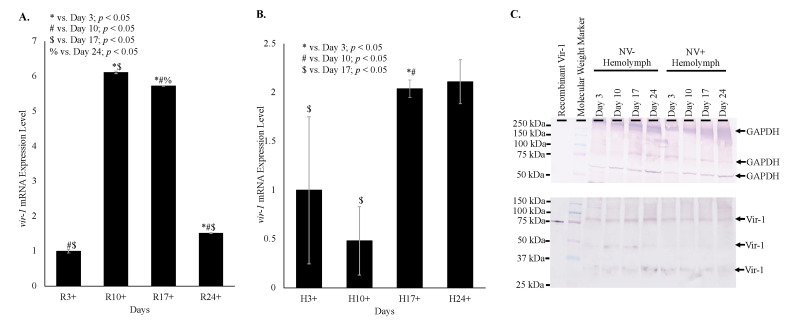
Vir-1 expression is increased in Nora virus-infected *D. melanogaster*. (**A**). qRT-PCR analysis of *vir-1* transcription in whole Nora virus-infected *D. melanogaster* on days 3, 10, 17, and 24 relative to age-matched virus-free controls. The results are from 3 experiments done in triplicate. The error bars are standard error of the mean, and ^*,#,$,%^ represent statistical significance (*p* < 0.05) for timewise comparisons as denoted in the figure legend. (**B**). qRT-PCR analysis of *vir-1* transcription in hemolymph of Nora virus-infected *D. melanogaster* on days 3, 10, 17, and 24 relative to age-matched virus-free controls. The results are from 3 experiments done in triplicate. The error bars are standard error of the mean, and ^*,#,$,%^ represent statistical significance (*p* < 0.05) for timewise comparisons as denoted in the figure legend. (**C**). Western blot analysis of time study NV+ and NV− hemolymph samples. The top panel is the control that used anti-GAPDH primary antibody for detection. Arrows indicate expected GAPDH products at approximately 300, 250, and 50 kDa. The bottom panel used anti-Vir-1 serum as the primary antibody for detection. Arrows indicate products at approximately 75, 50, and 30 kDa.

Despite Vir-1 presence in both virus-free and Nora virus-infected *D. melanogaster*, qRT-PCR data indicates increased transcription of *vir-1* in Nora virus-infected *D. melanogaster.* Transcription of *vir-1* in whole fly lysates increased 6-fold in day 10, 6-fold in day 17, and 1.5-fold in day 24 relative to day 3 Nora virus-infected *D. melanogaster* relative to age-matched virus-free controls. All ages were significantly different (*p* < 0.05) from each other (Figure 5A). Transcription of *vir-1* in hemolymph of Nora virus-infected *D. melanogaster* was downregulated 0.5-fold in day 10 and upregulated 2-fold in day 17 and day 24 relative to day 3 (Figure 5B). Differences between days 3 and 17 and days 10 and 17 were significant (*p* < 0.05). Despite increasing *vir-1* transcription, levels of Vir-1 protein remain stable in hemolymph across all time points by Western blot analysis. Performing quantification using ImageJ (https://imagej.nih.gov/.html) demonstrated no overall statistically significant increase of Vir-1 between NV+ and NV− samples, as well as between time points (Figure 5C).

## 4. Discussion

The current study provides evidence that Nora virus may be circulating in the hemolymph and inducing two candidate immune genes, *vir-1* and *vago*. Nora virus was previously identified primarily within the gut tissue of infected *D. melanogaster* [13]. After identifying a locomotor defect in infected *D. melanogaster* [15], it was hypothesized that Nora virus may circulate to other tissues by way of the hemolymph. Nora virus RNA for the *ORF1* gene and capsid protein, VP4b, were found to be present in the hemolymph of infected *D. melanogaster* both by Western blot and qRT-PCR (Figure 1). Presence of VP4b in the hemolymph of infected *D. melanogaster* indicates that viral capsid components are present in the hemolymph (Figure 1A), while qRT-PCR amplification of Nora virus *ORF1* in hemolymph samples (Figure 1C) supports the possibility of full virions circulating in the hemolymph. The lower concentration of *ORF1* transcript levels in the hemolymph compared to whole fly lysates is likely due to two factors. The first is that the site of Nora virus replication is in the midgut of the organism [12], which is where the highest levels of *ORF1* transcripts would be expected to be found. Second, the hemocytes in the hemolymph act similar to mammalian monocytes/macrophages in that they phagocytose and encapsulate invading pathogens, such as a virus [21]. Therefore, the lower viral titer in the hemolymph could be due to viral clearance by these cells. Viral circulation in the hemolymph of *D. melanogaster* has not previously been demonstrated. However, dissemination of arboviruses into the hemolymph is well-documented in mosquito vectors [22,23]. One study found that infecting viral particles may cross the midgut basal lamina into the mosquito hemocoel without replicating within the midgut tissue [23].

Overall, this data supports the possibility of Nora virus circulating in the hemolymph from the gut to the brain, contributing to the locomotor defect observed in infected *D. melanogaster*. Viral circulation through *Drosophila* hemolymph is not yet well studied. However, the proposed circulation of Nora virus would require the virus to overcome several obstacles. First, Nora virus must be able to cross the barrier of the gut lamina. Even after crossing the gut lamina, the virus must remain infectious and evade destruction by circulating hemocytes. Lastly, the virus must be capable of entering a secondary site and establishing an infection. Additional study of Nora virus circulation is warranted, investigating the infection of secondary tissues. The ability of Nora virus to invade and infect the brain and muscle tissue should be investigated to determine which secondary sites of infection might contribute to the motor defect phenotype. Other picorna-like viruses have been identified, which infect the nervous tissues of various insects, including the novel Kakugo virus, which infects the brain tissue of worker honeybees [24].

Nora virus is a novel RNA virus that appears to share many features with other RNA viruses. One of these features is the biphasic model of viral load (Figure 1C and Figure 2), which is seen in other picornaviruses, such as enterovirus. For example, the load of coxsackievirus B3 in infected mice also follows a biphasic model [25]. In this study, it was found that Nora virus shows a biphasic model of viral load in both whole fly lysates and in the hemolymph (Figure 1C and Figure 2). This suggests an initial period of rapid replication in the first 3 days of infection, quickly opposed by host production of antiviral proteins. As the immune response brings the viral load under control around Day 10 (Figure 1C and Figure 2), transcription of antiviral proteins decreased (Figure 3B). This allows the remaining virus to resume rapid replication, resulting in the late-life peak in viral load near day 24 (Figure 1C and Figure 2). Nora virus VP1 proteins have been shown to inhibit the RNAi pathway in vitro [26]. This inhibition or other unknown mechanisms may allow Nora virus to suppress the host anti-viral response.

The role of candidate immune system genes in response to Nora virus infection is of interest when determining how the virus is regulated. In previous studies, two genes upregulated by Nora virus, *vago* and *vir-1*, were identified [17,18], but not characterized. In one study, microarray analysis of Nora virus-infected *D. melanogaster* identified 46 upregulated and 12 downregulated genes. The study further investigated two of these genes, *Chorion protein 16* and *Troponin C isoform 4*, but left the remaining identified genes, including *vago*, for future analysis [17]. A later study used next generation sequencing to identify 35 upregulated immune response genes in Nora virus-infected *D. melanogaster* including *vir-1* [18]. In the current study, the production of Vago and Vir-1 were determined in response to Nora virus infection using a time course analysis, qRT-PCR and Western blot analyses. The data here show Vago was present (Figure 3A) and *vago* transcription was upregulated (Figure 3B,C) in response to Nora virus infection. Additionally, *vago* transcription was increased in the hemolymph of infected *D. melanogaster* (Figure 3B), following the viral load (Figure 1C). This suggests that Vago plays a role in the *D. melanogaster* immune response to Nora virus. This expression of Vago in response to viral infection was previously reported with DCV infection at day 4 post-infection. In addition, flies with mutant *vago* that were infected with DCV contained higher levels of virus than wild-type controls [9]. Therefore, the decreased level of virus in the hemolymph as compared to the whole body samples could be due to the increased expression of *vago.* Another study determined that Nora virus load was not impacted by *D. melanogaster* null mutations in RNAi components: *r2d2, AGO2,* or *Dicer-2*. It is proposed that Nora virus either disables the RNAi pathway or that replication occurs in a compartment inaccessible to the RNAi machinery, allowing the RNAi resistant replication of Nora virus [27]. The RNAi machinery is necessary for transcription of *vago*, as Relish requires TRAF-mediated dephosphorylation [8]. The increasing *vago* transcription demonstrated in this study suggests that the RNAi pathway is still functional in Nora virus-infected *D. melanogaster* and it is unlikely that Nora virus produces an RNAi inhibitor. This also supports the proposed replication compartment model, where the RNAi pathway continues to operate, promoting the transcription of *vago*, while allowing Nora virus replicates in an isolated compartment evading the effects of the RNAi machinery [13].

The role of Vago during viral infection is not well characterized and the data reported here is the first characterization of it during Nora virus infection. As previously mentioned, viral infection triggers expression of a number of genes, including *vago*, which encodes an 18 kDa polypeptide that may function either as an antiviral molecule targeting virions or as a cytokine stimulating an interferon-like response. The mechanism of how Vago is involved in establishing an immune response has been hypothesized as involving RNAi and JAK-STAT pathways. During infection with DCV, *vago* induction was dependent on Dicer-2, specifically the DExD/H-box helicase domain. This domain may act as a sensor that can induce *vago* transcription for an additional antiviral response. Further evaluation has shown similarities of the DExD/H-box helicase domain and the products of the RLR helicases of retinoic acid-inducible gene I (RIG-1), melanoma differentiation-associated protein 5 (MDA5), and laboratory of genetics and physiology 2 (LGP2) in mammals to sense viral RNA [9]. In mammals, these helicases respond to viral RNA by inducing interferon and secretions of interferons to stimulate antiviral activity in other cells, which provides further evidence for the role of Vago functioning as a cytokine. Another study evaluated the role of Vago during infection of *Culex* mosquitoes with the West Nile virus (WNV). During WNV, infection leads to secretion of Vago via Dicer-2 mediated stimulation of TRAF and Rel2, which leads to Rel2 inducing *vago* transcription. Once Vago is secreted, it activates the JAK-STAT pathway in neighboring cells to stimulate an antiviral response. This study found a proposed mechanism that involves Vago induction via Dicer-2 to stimulate an antiviral response via the JAK-STAT pathway. Again, this proposed pathway is similar to the RIG-1/TRAF/NF-kB interferon pathway in mammals [7]. However, NF-kB mediated induction of Vago in *Drosophila* was unable to be well studied, which suggests additional investigation to determine if the proposed mechanism in *Culex* is similar or an alternative mechanism exists for *Drosophila*.

As a proposed mechanism for Vago was developed, the interaction between Vago and vir-1 was further studied. Vir-1 has been implicated in antiviral defense, but its involvement is unclear and this is the first report of the Vir-1 response to Nora virus. During DCV infection, *vago*-mutant flies were created and *vir-1* was found to be fully inducible, which suggests Vago may not directly be involved in *vir-1* induction or may involve an alternative mechanism. In addition, this analysis was limited by the inability to evaluate the Vago protein as it was unstable. The authors concluded additional research would need to be conducted to determine the roles and interaction of Vago protein with Vir-1 [9]. In *Culex* mosquitos infected with WNV, *vir-1* was found upregulated via the JAK-STAT pathway indicating that Vago may play a role in inducing transcription of *vir-1* via the aforementioned proposed mechanism. In addition, vir-1 may also be induced by alternative unidentified virus-activated cytokines in absence of Vago expression in *Culex* and *Drosophila* [7]. As the antiviral defense mechanism for Vago is characterized, the role of Vir-1 may be better identified.

Time course data shows increasing transcription of *vir-1* in Nora virus-infected *D. melanogaster* as the Nora virus load increased (Figure 4). Increased transcription of *vir-1* has been demonstrated within 24 h after *D. melanogaster* infection with DCV [4]. Little is known about Vir-1, but the current study found a transition with age from high *vir-1* transcription in whole fly lysate early in life to increased transcription in hemolymph later in life (Figure 5). This may suggest a cytokine-like role of Vago in response to Nora virus infection. In this role, Vago may be produced and secreted by infected intestinal cells and act upon the JAK/STAT pathway of hemocytes. In this way, transcription of *vir-1* may increase in the hemolymph despite decreasing *vago* transcription levels by day 24. Production of another cytokine-like molecule, Upd3, has also been shown to be required for cytokine-like immune signaling in the hemolymph of septic *D. melanogaster* infected with *Escherichia coli* or *Micrococcus luteus* [28]. In this study, Vir-1 was found to be present in virus-free *D. melanogaster* (Figure 4 and Figure 5). This previously unidentified presence of Vir-1 may suggest baseline expression or an additional role of Vir-1. It is unlikely that this Vir-1 presence is due to infection with another virus as the stocks are regularly dechorionated and checked for the main *Drosophila* viruses. One study investigating the immune response in cold tolerance identified upregulation of *vir-1* in response to heat shock [11]. Further studies are needed to determine the function of Vir-1 in virus-free *D. melanogaster*, but this role may include a baseline expression level allowing for more rapid response to heat shock or viral infection.

## 5. Conclusions

As discussed previously, study in the *Drosophila* immune response to viral infection offers a model for mammalian immune responses and viral pathology. This study offers insight into two host immune proteins, Vago and Vir-1, which are not well understood. This may give rise to further study of similar proteins in mammalian immune responses. The data also offers insight into the pathogenesis of picorna-like viruses. This may allow for further modeling of picorna viruses through picorna-like viruses.

The current study found that Nora virus load follows a biphasic model in whole fly RNA and in hemolymph of infected *D. melanogaster*. The data supports the cytokine-like role of Vago in response to viral infection and suggests that Nora virus may replicate in a compartment inaccessible to the RNAi machinery. Additionally, *vir-1* transcription is found to be upregulated in response to Nora virus infection. However, the presence of Vir-1 in virus-free *D. melanogaster* suggests an additional role of Vir-1. This study demonstrated circulation of Nora virus in the hemolymph of infected *D. melanogaster* and biphasic viral load with *vago* and *vir-1* transcription levels mirroring this load.
